# Tubular Cellulose from Orange Juice By-Products as Carrier of Chemical Preservatives; Delivery Kinetics and Microbial Stability of Orange Juice

**DOI:** 10.3390/foods10081882

**Published:** 2021-08-15

**Authors:** Athanasia Panitsa, Theano Petsi, Panagiotis Kandylis, Maria Kanellaki, Athanasios A. Koutinas

**Affiliations:** 1 Food Biotechnology Group, Department of Chemistry, University of Patras, Rio, 26504 Patras, Greece; sissy_panitsa@hotmail.com (A.P.); thpetsi@upatras.gr (T.P.); M.Kanellaki@upatras.gr (M.K.); A.A.Koutinas@upatras.gr (A.A.K.); 2 Laboratory of Oenology and Alcoholic Beverages, Department of Food Science and Technology, School of Agriculture, Aristotle University of Thessaloniki, P.O. Box 235, 54124 Thessaloniki, Greece

**Keywords:** juice, sodium benzoate, *Saccharomyces cerevisiae*, *L. plantarum*, *L. casei*, delivery, orange pulp

## Abstract

The quality and safety of juices are assured mainly through heat treatments and chemical preservatives. However, there is a growing trend in the food industry for lowering energy and water demands, and the chemicals and additives that may have negative effects οn human health. Following that trend, in the present study, the reduced use of chemical preservatives in orange juice is proposed by using encapsulated sodium benzoate (SB) in tubular cellulose (TC), derived from orange pulp. The effects of SB concentration and contact time on SB encapsulation were evaluated. The use of the wet impregnation method, 12% *w/v* SB solution and 2 h of contact proved to be ideal for application in the juice industry. The use of starch gel resulted in a more stable composite (TC/SB-SG) with a slower SB delivery, showing its potential for future controlled delivery applications. Furthermore, similar delivery rates of SB in juice were noted at 25 and 2 °C. The TC/SB-SG proved capable of inhibiting the growth and reducing the numbers of spoilage microorganisms (yeasts and lactic acid bacteria). The results of the present study are promising for potential applications; however, more research is needed in order to evaluate the controlled delivery of SB in juice.

## 1. Introduction

The purpose of adding preservatives to food is the avoidance of microbial spoilage or alterations in their chemical composition and, therefore, the extension of their lifespan [1,2]. There are various preservatives that are widely used in the industry, and among them are benzoic acid and its salts. Due to their antimicrobial activity, they can be added to carbonated drinks, fruit juice, vinegar and, in general to foods and drinks with low pH values [1,3,4]. Sodium benzoate (SB) is among the most commonly used preservatives in food, and especially in fruit juices [1,2,4,5,6,7]. SB was the first preservative that the FDA allowed to be used in food products [6].

Fruits and fruit juices due to their high content of organic acids have, in most cases, low pH values (2.0–4.5). It is therefore more difficult for them to be spoiled by microorganisms. However, microbial degradation can occur when low-pH-resistant microorganisms grow, such as acid-tolerant bacteria (for example, lactic, acetic and propionic bacteria), yeasts (such as sugar-fermenting yeasts) and molds [8,9]. Such alterations may lead to creation of off-flavor compounds, discoloration, changes in texture and appearance; thus, the products are distorted and become unsuitable for commercial use [8]. In addition, microorganism spoilage can cause the production of carbon dioxide that may lead to distortion or leakage of cans or bottles. Furthermore, several cases of microbial contamination by pathogens in foods such as fruit juices have been reported. Microorganisms such as *Escherichia coli* O157:H7, *Salmonella* and the parasite *Cryptosporidium* can be transmitted from raw fruit juice, causing several illnesses [8,9].

However, despite its effective antimicrobial activity, SB also has some side effects on the human body. For example, it can burden the liver, cause sensitization and affect children’s behavior. The mutagenic and cytotoxic effects of SB are proven in vitro on human lymphocytes, and they may cause cancer [2]. SB is metabolized within the mitochondria, producing hippurate, which is then cleared by the kidneys. Ingestion of SB at doses generally regarded as safe (GRAS) can lead to a high increased hippurate level in the plasma. Side effects of benzoate and hippurate on glucose homeostasis in cells and animal models have been reported [5]. Beverages with benzoate salts contain low levels of the carcinogen benzene, which may be formed during storage after decarboxylation of the benzoate through hydroxyl radicals [10].

In order to avoid excessive quantities of SB in food, an intermediate carrier, in which SB can be encapsulated and released gradually, can be used. This work aims at the preparation of a suitable material for the encapsulation of SB and its gradual release in drinks such as orange juice. Although the addition of SB in fresh juices is continuously reducing, in carbonated and non-carbonated soft drinks based on juices, SB is necessary. Tubular cellulose (TC) is a natural material with great potential as an encapsulation/immobilization carrier. In several studies, TC from various origins has already been used successfully for the encapsulation of enzymes (TC from wood sawdust) [11] and microbial cells (TC from Indian mango, sal wood and rice husk) [12] and for the preservative delivery in meat products (TC from leaf celery and spinach) [13]. Following this trend, the aim of the present study is the evaluation of TC from edible orange pulp as a suitable material for the delivery of SB in orange juice. Encapsulation of SB could lead to (i) delay of the delivery of increased amounts of SB in juices, (ii) maintenance of its antimicrobial activity and (iii) reduction in SB side effects on human health. Furthermore, the similar nature of orange pulp to that of orange juice is expected to have no effect on the characteristics of the final product.

## 2. Materials and Methods

### 2.1. Raw Materials, Microorganisms and Chemicals

Navelina oranges were purchased from a local market. The oranges were squeezed and the pulp and orange peel pith were kept for treatment and the encapsulation of SB.

For the delivery of SB, concentrated orange juice was obtained from the local industry (Loux S.A., Patra, Greece) and after appropriate dilution was used in the delivery of the preservative experiments (23 g/100 mL sugars, pH 4.27, total acidity 12.8 g citric acid/L, volatile acidity 0.08 g acetic acid/L). 

NaOH pellets were obtained from Lachner, food grade SB was purchased from Sigma Aldrich, and food grade corn starch was purchased from Wintersun Chemical.

For the microbial stability of juices, commercial orange juice purchased from a local market was used (*Μαράτα*, natural orange juice, Sklavenitis Group, Athens, Greece). Its composition per 100 mL was as follows: 9.0 g sugars, 0.6 g edible fibers, 0.6 g proteins, 0 g fats, 0 g salt and 40 mg vitamin C. The following microorganisms were used: *Lacticaseibacillus* (former *Lactobacillus*) *casei* ATCC 393 (DSMZ, Braunschweig, Germany), *Lactiplantibacillus plantarum* 2035 (from the collection of the Laboratory of Food Microbiology and Hygiene, Department of Food Science and Technology, Aristotle University of Thessaloniki, Greece), *Saccharomyces cerevisiae* CR51 (Mangrove Jacks) and *Saccharomyces cerevisiae* EC1118 (Lalvin, Lallemand Brewing).

### 2.2. Tubular Cellulose from Orange Pulp

Delignification was carried out by the treatment of orange pulp with 1% *w/v* NaOH and heating at 70 °C for 3 h (Table 1) [13]. The delignified orange pulp (TC) was dried for approximately 48 h, at −45 °C (temperature of condenser) under vacuum pressure of 15 × 10^−3^ mbar using the freeze-drying process (Labtech Freeze Dryer, LFD 5508, Daihan Labtech Co., Ltd, Namyangju, South Korea).

The surface characterization of orange pulp was performed using a Micromeritics Tristar surface area and porosity analyzer and scanning electron microscope as described previously [13].

### 2.3. Sodium Benzoate Encapsulation

Encapsulation of SB using the wet impregnation method (WI) (TC/SB-WI) was conducted by adding TC in SB solution with continuous stirring. In order to study the effect of contact time on the final SB amount encapsulated, 30 mL of 15 and 30% *w/v* SB solution was added to 1.5 g TC, and samples were taken at 0.5, 2, 3.5 and 5 h. In order to study the effect of initial concentration of SB solution, different concentrations of SB (1.5–49% *w*/*v*) were prepared in 30 mL of deionized water, and 1.5 g TC was added. The mixtures were left under stirring for 2 h. 

TC/SB-SG (tubular cellulose/sodium benzoate substrates using the starch gel method (SG)) was prepared following the method described by Panitsa et al. [13] with slight modifications (7 mL of deionized water was mixed with corn starch and 5% *w/v* SB solution).

### 2.4. Sodium Benzoate Delivery in Orange Juice

Two grams of freeze dried TC/SB-WI and TC/SB-SG (containing 0.13 g of encapsulated SB) was placed in 100 mL of juice. Several samples (100 mL each) were placed at 2 ℃ and at room temperature (25 °C) without stirring for up to 60 days. In several time intervals (up to 60 days), a sample was taken for SB analysis.

### 2.5. Microbiological Stability

*L. casei*, *L. plantarum* and *S. cerevisiae* were activated prior to their usage in the contamination experiments based on the method described by Katsaros et al. [14] with some modifications. The two strains of lactic acid bacteria from glycerol stock cultures were inoculated into MRS broth tubes and incubated at 30 °C for *L. plantarum* and 37 ℃ for *L. casei* for 24 h [15,16]. After activation, 100 μL of each was transferred into the MRS broth and was incubated at the same temperatures for 24 h. Activation of the two strains of yeasts from freeze-dried cultures was performed by inoculating cells into a yeast extract peptone glucose broth (YPGB) composed of (*w*/*v*) 0.5% yeast extract, 0.1% peptone and 2.0% glucose, at 25 °C for 48 h. After activation, 100 μL was also transferred into the YPGB and incubated at 25 °C for 24 h. Additional enrichment of the cultures (lactic acid bacteria and yeasts) was performed, following the same procedure and incubation for 18 h–20 h.

Contamination of the juices was performed by transferring 1 mL of each microorganism to 100 mL of juice, separately, with and without encapsulated SB (0.13 g of SB). They were stored at 5 °C for up to 15 days. 

Juice samples were taken at 2, 5 and 15 days after contamination and serial diluted in Ringers’ solution (1/4 strength); then, solid cultures were prepared on MRS agar for *L. casei* (incubated at 37 ℃ for at least 48 h) and *L. plantarum* (incubated at 30 ℃ for at least 48 h) and on potato dextrose agar for *S. cerevisiae* (incubated at 30 ℃ for at least 72 h).

### 2.6. Chemical Analyses

The pH was measured directly in the juice sample with an automatic electronic pH meter (SensoDirect pH 110, Aqualytic (Lovibond), Dortmund, Germany). 

Total acidity (expressed as g of citric acid/L juice) was measured by the titration of 10 mL of orange juice with 0.1 M NaOH solution and phenolphthalein as the indicator. 

Volatile acidity (expressed as g of acetic acid/L juice) was determined after the distillation of 50 mL of juice. More specifically, 200 mL of distillate was collected and titrated with 0.1 M NaOH solution and phenolphthalein as the indicator.

The determination of sugars (sucrose, glucose and fructose) was performed using high-performance liquid chromatography (HPLC). A Shimadzu chromatograph was used, consisting of an LC-9A high-pressure pump, a Nucleogel Ion 300 OA column (300 × 7.8 mm id, 10 μm particle size), a CTO-10A oven, an RID-6A refractive index detector connected to a C-R6 integrator and a DGU-2A degassing unit. The analysis was performed using isocratic separation at 33 ℃ with a mobile phase of 0.017 N H_2_SO_4_ and a flow rate of 0.55 mL/min. Ten milliliters of juice was added to a 100 mL volumetric flask and filled with deionized water. From this solution, 500 μL was placed in a 25 mL volumetric flask, 500 μL of propanol-2 1% *v*/*v*, was added as an internal standard, and ultra-pure water was added. The samples after the above preparation were filtered with a 0.22 μm microfilter and a volume of 40 μL was injected into the chromatograph. The sugar concentrations were determined using standard curves.

The determination of SB was performed using high-performance liquid chromatography (HPLC). A Shimadzu Corporation chromatograph was used, consisting of a high pressure pump LC-20AT, a Supelco Discovery C8 column (250 × 4.6 mm id, 5 μm particle size), a CTO-10AC oven and an array detector of an M20A-DGA-single-diode SGD-A unit. The analysis was performed using isocratic separation at 30 °C with mobile phase 80% acetate buffer/20% acetonitrile, a flow rate of 0.8 mL/min and an SIL-20AC sampler. Five milliliters of each sample was placed in a 50 mL volumetric flask, and it was filled with 50% aqueous acetonitrile (solution 1). Ten milliliters of solution 1 was placed in a 50 mL volumetric flask and filled with 50% aqueous acetonitrile solution (solution 2). Then, 1 mL of solution 2 was mixed with 5 mL of mobile phase (solution 3). Then, 1 mL of solution 3 was mixed with 10 mL of mobile phase (solution 4). Solution 4 was centrifuged for 15 min at 4000 rpm, the supernatant was filtered through a 0.22 μm microfilter, and 5 μL from each sample was loaded on the chromatograph. SB was determined using standard curves.

### 2.7. Statistical Analysis

Duplicate experiments were conducted, and duplicate or triplicate samples were used for the analyses. Experimental data were evaluated for their significance (*p* < 0.05, 0.01 and 0.001) with analysis of variance (ANOVA) and Tukey’s honest significant difference (HSD) test using Statistica version 12.0 (StatSoft Inc., Tulsa, OK, USA). 

## 3. Results and Discussion

### 3.1. Delignification and Characterization of Orange Pulp

The first step of the present study was the preparation of an appropriate support for the encapsulation of chemical preservatives for applications in the food industry. In order to achieve an increased and sufficient encapsulation, the support should have the appropriate properties to guarantee this encapsulation. Delignification has been proven an ideal method for the pretreatment of lignocellulosic materials prior to the enzymatic process and hydrolysis, since the removal of lignin increases the available surface area of material and also increases the pore volume [17,18]. However, delignified materials have also been successfully used for the immobilization of microorganisms for several food applications [12,19,20]. Recently, our team reported the preparation of an edible tubular cellulose-based support for the delivery of chemical preservative in meat products [13]. Following that trend, in the present study, orange juice by-products such as orange pulp (orange pulp and orange peel pith) were delignified and evaluated as support for SB for applications in the juice and beverage industry. Delignification resulted in more than a two-times increase in specific surface area, a five-times increase in pore volume and a two-times increase in pore diameter (Table 2). These results are lower than those reported in the previous study with TC from leaf celery and spinach [13], which is attributed to the different composition of each material.

### 3.2. Preparation of Tubular Cellulose/Sodium Benzoate (TC/SB) Substrates

**Figure 1:**
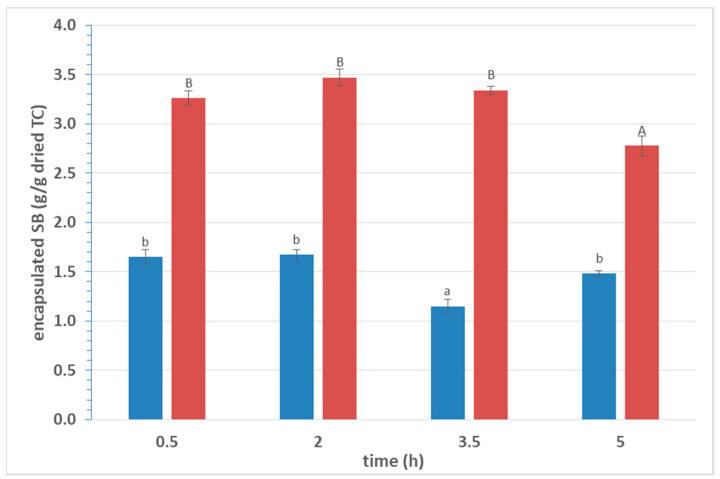
Effect of contact time on encapsulation of SB in dried TC. (SB: sodium benzoate; TC: tubular cellulose; (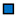) 15% *w/v* SB; (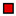) 30% *w/v* SB; means within the same SB concentration with different superscripts differ significantly (*p* < 0.05)).

The encapsulation of SB in TC was achieved by adding dried TC to a SB solution. In order to increase the rate of encapsulation, stirring was applied. The effect of contact time on encapsulation efficacy was not significant (*p* > 0.05) in the case of 30% *w/v* SB solution up to 3.5 h, while a reduction was observed in 5 h (Figure 1). Similar results were noted in the case of 15% *w/v* SB solution. For this reason, 2 h was selected as the ideal time of stirring/contact in order to ensure a high encapsulation rate.

**Figure 2:**
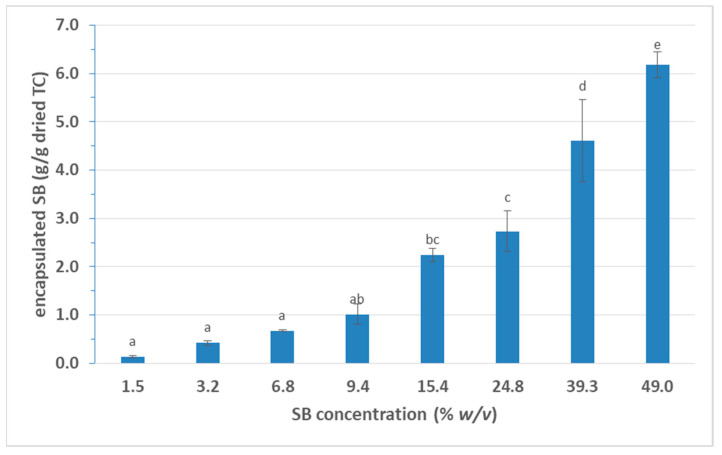
Effect of SB concentration on encapsulation of SB in dried TC. (SB: sodium benzoate; TC: tubular cellulose; means with different superscripts differ significantly (*p* < 0.05)).

Another important factor for the encapsulation of SB in TC was the concentration of the SB solution used. The results of Figure 2 clearly report that the increase in concentration of the SB solution resulted in a proportional increase in the encapsulated SB on the TC. More specifically, SB concentrations up to 9% *w/v* resulted in similar values (*p* > 0.05), while in higher than 9% *w/v*, the encapsulated SB was significantly higher (*p* < 0.05). Indeed, an encapsulation of higher than 6 g/g dried TC can be achieved using a 49% *w/v* SB solution. This proportional increase in the encapsulation ratio with the SB solution used is important since it is possible to prepare TC/SB composites with different concentrations of SB for different applications in the food industry. 

### 3.3. Sodium Benzoate Delivery 

The effect of the encapsulation method, temperature and time on SB delivery in orange juice is presented in Table 3. The effect of temperature was significant mainly after 360 h. This observation may be important since the delivery of SB is the same even at low refrigerator temperatures, which will also help the increased shelf life of the juice. Regarding the effect of encapsulation method, the use of SG seems more appropriate since slower SB delivery is achieved. The presence of SG strongly affected the SB release, thus suggesting the potential of SG encapsulation to control the release mechanism of active compounds, as was also reported in the case of alginate beads [21]. This may be attributed to the better encapsulation of SB and to the starch gel matrix that permits low SB mobility. However, both encapsulation methods could have practical use depending on the rate of SB delivery required. 

### 3.4. Antimicrobial Effectiveness of TC/SB Susbstrates in Orange Juice

The quality and safety of juices is assured mainly through heat treatments due to their effectiveness and traditional nature. However, these treatments usually have negative impacts on the final nutritional quality and fresh-like characteristics of the juice. In order to overcome such problems, several non-thermal approaches have been evaluated in recent years [22]. Furthermore, there is a growing trend in the food industry for lowering energy and water demands, and the chemicals and additives that may have negative effects on human health, so-called “green consumerism” [23]. Following this trend, in the present study, the reduced use of chemical preservatives in orange juice is proposed by using TC/SB-SG. Its effectiveness was evaluated after contamination of orange juice with yeasts and lactic acid bacteria, and the results are presented in Table 4. 

*Saccharomyces cerevisiae* is a common spoilage yeast in juices; therefore, it is usually used in studies with contaminated juices [24,25,26,27,28,29]. In the case of lactic acid bacteria, *L. plantarum* and *L. casei* are also reference spoilage strains used in orange and other juices [26,27,30,31]. Although relatively high initial contamination levels were selected (8.3 log CFU/mL in lactic acid bacteria and 7.4 log CFU/mL in yeasts), the encapsulated SB resulted in significantly lower contents compared to the control juice. SB was more effective in the case of yeasts during the first days of storage (2–2.5 log CFU reduction). After the 5th day and up to 15th day, the reduction was the highest in all cases due to the gradual release of SB in the orange juice. These results are promising considering the fact that the usual levels of contamination are significantly lower than those tested in the present study. However, more research is needed in order to evaluate more microorganisms and the possibility to control the release of SB.

## 4. Conclusions

A new method for the potential controlled delivery of SB in juices was reported by using encapsulation. The encapsulated methods that were evaluated resulted in different rates of SB delivery, while the use of different SB solutions gave different encapsulation rates. All of these provide a variety of TC/SB composites with different potential application in the food industry. The use of the WI method, 12% *w/v* SB solution and 2 h of stirring/contact seems ideal for application in the juice industry. However, the use of starch gel resulted in a more stable composite (TC/SB-SG) with slower SB delivery. The delivery proved capable of inhibiting the growth of potential spoilage microorganisms and even significantly reducing their numbers. Although the results are promising for potential applications, more research is needed in order to evaluate the controlled delivery of SB in juice or similar matrices and the possible effects on the organoleptic (flavor, aroma and color) properties of the product.

## Figures and Tables

**Table 1:** Experimental design.

**1. Tubular Cellulose (TC) from Orange Pulp**
Orange pulp (pulp and peel pith) → delignification (1% *w/v* NaOH + 70 °C for 3 h) → TC (freeze drying; FD)
**2. Sodium Benzoate Encapsulation**
I. TC/SB-WI (Wet Impregnation Method)
TC (1.5 g) + SB solution (30 mL; 1.5–49% *w*/*v*) + stirring (0.5–5 h)
II. TC/SB-SG (Starch Gel Method)
7 mL of deionized water was mixed with corn starch and 5% *w/v* SB solution + TC
**3. Sodium Benzoate Delivery in Orange Juice**
2 g FD TC/SB-WI and FD TC/SB-SG (containing 0.13 g of encapsulated SB) + 100 mL orange juice
Delivery after 0.5–5 h and 0.5–60 days; 2 °C and 25 °C
**4. Microbiological Stability**
Contaminated juice with *L. casei*, *L. planatrum* and *S. cerevisiae* (storage 0, 2, 5 and 15 days; 5 °C)

**Table 2:** Porosimetry analyses of untreated and delignified orange pulp.

Analyses	Untreated Orange Pulp	Delignified Orange Pulp
Specific surface area (BET ^1^) (m^2^/g)	0.30 ± 0.03	0.67 ± 0.05
Pore volume (BJH) (×10^−3^ cm^3^/g)	1.3 ± 0.4	6.7 ± 0.5
Average pore diameter (BET) (Å)	47.0 ± 5.6	115.3 ± 13.2

^1^ BET: Brunauer–Emmett–Teller; BJH: Barrett–Joyner–Halenda.

**Table 3:** Effect of time on sodium benzoate delivery (%) in orange juice at 2 °C and 25 °C.

Time (h)	2 °C		25 °C		Significance	
	SG	WI	SG	WI	WI/SG	Temperature
0.5	10.6 ± 4.2 ^a^	9.9 ± 2.1 ^a^	16.9 ± 0.2 ^a^	9.0 ± 0.6 ^a^	ns	ns
2	12.0 ± 0.5 ^ab,A^	16.6 ± 1.2 ^ab,B^	16.2 ± 1.2 ^a,AB^	16.3 ± 1.4 ^ab,AB^	*	ns
5	17.9 ± 2.7 ^abc^	17.9 ± 2.1 ^abc^	18.8 ± 2.1 ^ab^	18.7 ± 3.4 ^bc^	ns	ns
12	17.3 ± 5.7 ^abc^	18.3 ± 1.4 ^abc^	17.6 ± 0.3 ^a^	21.0 ± 1.1 ^bcd^	ns	ns
24	17.8 ± 0.7 ^abc,A^	19.3 ± 0.5 ^bc,AB^	20.9 ± 0.7 ^ab,AB^	24.5 ± 1.2 ^bcde,B^	*	*
48	18.4 ± 1.5 ^abc,A^	25.4 ± 1.5 ^cde,B^	21.8 ± 0.7 ^ab,AB^	25.8 ± 0.7 ^cde,B^	**	ns
72	19.3 ± 0.9 ^abc^	24.4 ± 2.8 ^bcd^	21.2 ± 3.7 ^ab^	25.9 ± 0.7 ^cde^	*	ns
96	21.9 ± 0.7 ^bcd^	25.8 ± 0.7 ^cde^	24.9 ± 3.5 ^abc^	26.0 ± 0.9 ^cde^	ns	ns
120	20.9 ± 2.0 ^abcd,A^	28.4 ± 1.4 ^de,B^	26.0 ± 0.1 ^abc,AB^	28.4 ± 1.4 ^de,B^	**	ns
240	21.9 ± 0.7 ^bcd^	28.6 ± 3.8 ^de^	28.1 ± 1.8 ^bcd^	31.0 ± 2.3 ^ef^	*	ns
360	23.5 ± 2.2 ^cd,A^	29.5 ± 3.1 ^de,AB^	32.8 ± 4.5 ^cde,AB^	39.2 ± 3.5 ^fg,B^	ns	*
720	27.6 ± 5.2 ^cd^	34.0 ± 2.8 ^ef^	37.1 ± 3.5 ^de^	41.8 ± 3.5 ^g^	ns	*
1440	31.5 ± 0.7 ^d,A^	38.2 ± 2.1 ^f,AB^	40.7 ± 3.3 ^e,AB^	48.2 ± 4.5 ^g,B^	*	*

^a–g^ Means within a column with different lowercase superscripts differ significantly (*p* < 0.05); ^A–B^ means within a row with different uppercase superscripts differ significantly (*p* < 0.05); SG: tubular cellulose/sodium benzoate substrates by starch gel method; WI: tubular cellulose/sodium benzoate substrates by wet impregnation method; ns: not significant; * *p* < 0.05; ** *p* < 0.01.

**Table 4:** Effect of encapsulated sodium benzoate (TC/SB-SG) on inactivation of spoilage microorganisms (log CFU/mL) of orange juices during 15 days of storage at 5 °C.

Time (d)	*L. casei*		*L. plantarum*		*S. cerevisiae*			
					EC1118		CR51	
	C	SB	C	SB	C	SB	C	SB
0	8.31 ± 0.01 ^ab^	8.31 ± 0.01 ^a^	8.32 ± 0.01 ^a^	8.32 ± 0.01 ^a^	7.41 ± 0.04 ^ab^	7.41 ± 0.04 ^a^	7.38 ± 0.03 ^ab^	7.38 ± 0.03 ^a^
2	7.69 ± 0.01 ^b^	7.68 ± 0.68 ^a^	8.54 ± 0.06 ^b^	7.69 ± 0.34 ^a^	7.49 ± 0.02 ^bc^	4.75 ± 0.38 ^b^	7.54 ± 0.01 ^a^	5.57 ± 0.03 ^b^
5	8.35 ± 0.07 ^ab^	7.35 ± 0.01 ^a^	8.63 ± 0.06 ^b^	7.48 ± 0.02 ^a^	7.66 ± 0.05 ^c^	2.78 ± 0.67 ^c^	7.59 ± 0.12 ^a^	3.93 ± 0.11 ^c^
15	9.97 ± 1.07 ^a^	1.05 ± 0.07 ^b^	8.34 ± 0.03 ^a^	5.70 ± 0.28 ^b^	7.30 ± 0.06 ^a^	1.70 ± 0.28 ^c^	7.19 ± 0.02 ^b^	4.00 ± 0.07 ^c^
Significance	ns	***	**	**	**	***	*	***

^a–c^ Means within the same microorganism with different lowercase superscripts differ significantly (*p* < 0.05); C: control orange juice; SB: orange juice with encapsulated sodium benzoate; ns: not significant; * *p* < 0.05; ** *p* < 0.01; *** *p* < 0.001.

## Data Availability

All data are contained within the article.
